# Patients’ daily reporting of symptoms via mobile application reveals a significant difference between patients’ perceptions and doctors’ interpretations

**DOI:** 10.3389/fonc.2025.1595322

**Published:** 2025-07-08

**Authors:** Cvetka Grašič Kuhar, Nina Privšek, Marjetka Sraka, Ema Grašič, Timotej Kovač, Matjaž Kukar

**Affiliations:** ^1^ Department of Medical Oncology, Institute of Oncology Ljubljana, Ljubljana, Slovenia; ^2^ Faculty of Medicine Ljubljana, University of Ljubljana, Ljubljana, Slovenia; ^3^ Faculty of Medicine Maribor, University of Maribor, Maribor, Slovenia; ^4^ Faculty of Computer and Information Science, University of Ljubljana, Ljubljana, Slovenia

**Keywords:** patient-reported outcome, mobile application, breast cancer, chemotherapy, symptoms

## Abstract

**Purpose:**

Electronic patient-reported outcomes (ePROs) are gaining importance. The aim of this study was to investigate the difference in the reporting of symptoms between patients via mobile application (m-app) and doctor assessments. Additionally, usability and satisfaction with the use of the m-app were assessed.

**Methods:**

In this single-center prospective cohort study, we analyzed ePROs in 46 patients receiving (neo)adjuvant chemotherapy for early breast cancer. Patients recorded the occurrence and intensity of symptoms via the Android-based m-app daily. Three-monthly, patients completed validated quality of life questionnaires (EORTC C30 and BR23). For the 10 most common symptoms reported by patients, we compared the frequencies and grades between patients and doctors. Additionally, we compared daily symptom reports with questionnaire results. Finally, the usefulness of and level of satisfaction with the m-app by patients and doctors were evaluated.

**Results:**

During the study, patients recorded almost twice as many different symptoms through the m-app as doctors did in the electronic health records (75 *vs* 49). Symptoms were described by patients as mild (67%), moderate (30%), or severe (3%). The frequency and intensity of symptoms reported by patients were significantly higher than those reported by doctors (p<0.001). Fatigue, insomnia and dry mouth were the three main symptoms reported in more than 75% of patients, but insomnia and dry mouth were also the symptoms most often underreported by doctors. After three months of chemotherapy, QoL assessments revealed worsening of physical, social, cognitive and role functioning, increased fatigue, systemic therapy side effects, and dyspnea but a reduction in arm and shoulder problems. Patients and doctors rated the m-app with an overall score of 4.5 out of 5 (IQR 1.0). Patients expressed high levels of satisfaction with and usability of the m-app. In contrast, doctors were somewhat reluctant to perceive ePROs as an additional burden with patient management.

**Conclusion:**

This study revealed a significant discrepancy between patients’ daily symptom reports via the m-app and doctors’ assessments, indicating that healthcare professionals may not fully capture patients’ experiences. This underscores the importance of integrating PROs to accurately evaluate patients’ conditions. Our m-app serves as a promising tool for reporting and managing side effects during systemic treatment of breast cancer.

## 1 Introduction

Breast cancer is the most common cancer among women in Slovenia. In 2019, the age-standardized incidence rate was 108.4 per 100,000 women according to the European standard population (1). Over time, the net survival of breast cancer patients in Slovenia has improved significantly, from 77.5% (95% CI 76–79) during 1997–2001 to 87.6% (95% CI 86.3–88.9) during 2011–2015 (2). While survival rates have improved, maintaining quality of life (QoL) during cancer treatment is increasingly prioritized in clinical trials and real-world practice. Enhancing overall survival (OS) or QoL is a key endpoint for the regulatory approval of new therapies (3).

The evaluation of QoL covers different aspects of an individual’s daily life, including physical and emotional well-being, psychosocial functioning and treatment-related toxicity. Information about a patient’s health that comes directly from the patient without the intervention or interpretation of a healthcare professional are patient-reported outcomes (PROs) (4). PROs are assessed via patient-reported outcome measures (PROMs), usually with the Patient-Reported Outcomes version of the Common Terminology Criteria for Adverse Events (PRO-CTCAE) (5) or validated questionnaires. The European Organization for Research and Treatment of Cancer (EORTC) has developed three primary types of questionnaires to evaluate patient well-being: core questionnaires, module questionnaires, and standalone questionnaires. Core questionnaires serve as a basis for assessing quality of life in all cancer patients, whereas module questionnaires aim to increase sensitivity and specificity in specific types of cancer (6). The most commonly utilized PROMs in breast cancer include the EORTC Quality of Life Core Questionnaire 30 (EORTC QLQ C-30) and EORTC Breast Module 23 (EORTC QLQ BR-23) (7, 8). The impact of using PROMs to improve QoL has been demonstrated in several clinical trials, which revealed that early detection of adverse events not only increased QoL but also reduced the hospitalization rate and even prolonged OS (9–14). However, the main limitation of most existing methods of data collection is the recording of data on predefined dates (e.g., at clinic visits, every 12 weeks), which leads to information gaps between assessments and the use of limited sets of preselected symptoms that cannot capture all that actually occurred during therapy. Advances in informatics and mobile technology have enabled electronic patient-reported outcomes (ePROs) in real time via mobile applications (m-apps), offering a potential solution to overcome these limitations. Because the use of PROs even prolongs OS, ePROs are a way to become digital therapeutics (15). The use of the m-app with the PRO-CTCAE as a tool for self-reporting significantly empowers patients to adequately report adverse events of cancer chemotherapy and increases their self-management of treatment-related side effects (16–18). Despite their technological feasibility, the widespread adoption of such m-apps remains limited, probably due to the challenges in managing severe symptoms and physicians’ reluctance to accept new technologies (19).

To investigate the feasibility and utility of ePRO collection with our custom-made m-app OnkoVed (20), we conducted a prospective cohort study in patients receiving chemotherapy. Our primary objective was to assess the frequency and severity of symptoms reported by patients on a daily basis. We also assessed QoL via validated QLQ questionnaires within the m-app. In addition, we investigated the concordance between patient-reported and doctor-reported symptoms. Finally, we evaluated the usability and satisfaction of the m-app by patients and doctors.

## 2 Patients and methods

### 2.1 Study design

A single-center prospective study was conducted at the Institute of Oncology Ljubljana to assess the frequency and severity of symptoms occurring daily during chemotherapy for solid cancer. Patients received access to m-app OnkoVed, which includes a symptom collection module and the EORTC QLQ C30 and QLQ BR23 electronic questionnaires. In the present study, we report the results for patients with early breast cancer. The duration of the study was limited to the duration of (neo)adjuvant treatment (4–6 months). Patient accrual took place from April 2021 to July 2022. The study protocol was approved by the institutional review board and the ethics committee (ERIDNPVO-0003/2021 and ERIDNPVO-0011/2023). All patients signed an informed consent form before inclusion in the study. The study was conducted in accordance with the Declaration of Helsinki and Good Clinical Practice.

### 2.2 Patients

The inclusion criteria were as follows: age ≥ 18 years, early breast cancer (stage I-III), planned treatment with neoadjuvant or adjuvant chemotherapy (and anti-HER2 therapy in case of HER2 positivity), owner of an Android smartphone, and experience in smartphone use. The exclusion criteria were metastatic disease, owner of a non-Android smartphone, not proficient in smartphone usage or not understanding the Slovenian language.

### 2.3 Development of the mobile application OnkoVed

On the basis of our previous experience with m-app mPRO Mamma (21), which was designed only for patients with breast cancer, we made an upgrade to m-app OnkoVed to be suitable for all cancer patients receiving systemic therapy (20). This application contains a collection of all symptoms described in the PRO-CTCAE (5). If any symptoms occurred, the patient was required to record them and indicate their severity on a three-point scale as mild, moderate or severe. If there were no symptoms, no action was needed. In addition, electronic versions of the QLQ C30 and BR23 were added. At predetermined timepoints, patients sent data to the Institute of Oncology.

### 2.4 Study procedures

**Figure 1:**
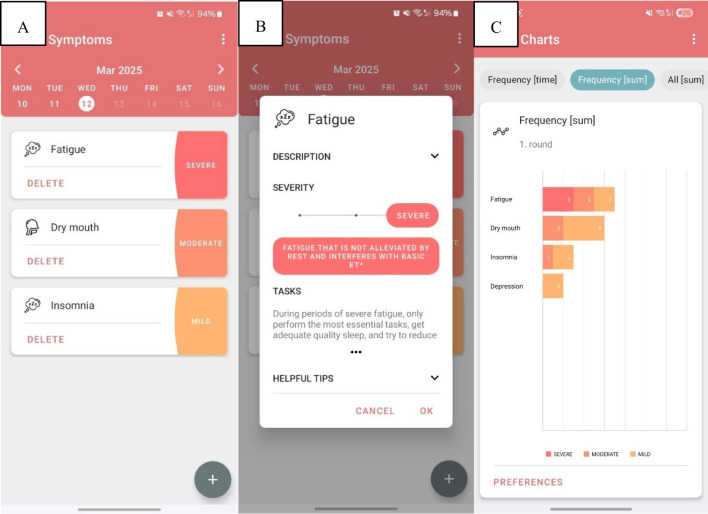
Screenshot of the mobile application showing patient-recorded symptoms with their grades **(A)**. Screenshots of the description of symptom fatigue and helpful tips for self-managing this symptom **(B)** and graphical presentation of symptoms (their grades and duration) **(C)**.

Upon enrollment in the study, patients received written instructions for using the m-app OnkoVed. They proceeded by downloading the application to their smartphones via the Google Play Store. As the application is publicly available and certain features are meant to be used by other interested parties, an activation of study-specific features (i.e. symptom sharing) was needed, which could be performed via the credentials provided in the instructions. The study nurse demonstrated to each participant how to use the application and send the reports. The m-app contains modules on patients’ demographic and cancer data, a symptom module (**Figure 1A**) with relevant tasks and useful tips according to the severity of a symptom (symptom tool-kit) (**Figure 1B**) and their cumulative frequency (**Figure 1C**), a module with the QLQ C30 and QLQ BR23 questionnaires, and a module with useful links (visiting planners, booklets, and cancer patients’ associations). After the account was activated, patients independently configured daily in-app notifications for symptom logging at their preferred times and could retrospectively enter symptom data for the preceding five days, with the primary impetus for daily app usage resting with the patient. Additionally, three-monthly notifications for answering QLQ C30 and QLQ BR23 were created. At the end of each chemotherapy cycle, a day before the next scheduled visit, the patient was required to send an e-PRO report. All collected data were transmitted in a secure encrypted format to a dedicated e-mail address created and maintained by the Institute of Oncology. With respect to patient engagement, the study nurses provided a phone reminder for daily symptom reporting only if a patient had not submitted their report the day prior to a scheduled check-up, which occurred every two to three weeks; the exact frequency of these calls was not recorded. Patients were not contacted if there was no interaction with the app.

At the scheduled appointment with the doctor, the study nurse provided the printed ePRO report received from the patient to the doctor. The application generated reports in three formats: free text, graphical presentation and an Excel table. In the free text, data concerning all symptoms were reported as follows: day/cycle/symptom type/grade (**Supplementary Figure 1**). In the graphical presentation, time course display of the symptoms with their grades were highlighted in different colors (**Supplementary Figure 2**). Finally, all symptoms were presented on an Excel table, which was suitable for statistical analysis (**Supplementary Figure 3**).

Doctors were informed that the ePRO app featured a list of 80 patient-adapted symptoms based on the PRO-CTCAE framework and that the system generated comprehensive reports in text, graphical, and spreadsheet formats. While the intention was for doctors to review these reports, discuss them with patients, and report them in the patient’s electronic health record (EHR), most continued with their standard consultation practices, prompting a retrospective analysis comparing patient-reported symptoms with doctor documentation.

One of the doctors performed retrospective grading of all doctors’ reporting of symptoms according to the Common Terminology Criteria for Adverse Events v5 (22) at the end of the study. Doctors’ reported symptoms (at check-ups) were compared with the daily reports of patients. We were aware of the possibility of assessment bias in the doctor’s assessment of symptom grade due to the retrospective nature of the assessment.

### 2.5 Study endpoints

The primary outcome was the frequency and severity of patient-reported symptoms during chemotherapy on the basis of daily m-app records and doctor reports in EHRs during outpatient visits. We specifically focused on the ten most frequently reported symptoms and compared the concordance of symptom frequency and grades reported by patients and doctors. The duration of symptoms was also assessed. The secondary outcome was the change in QoL as assessed by the EORTC QLQ-C30 and the QLQ-BR23. Finally, we assessed the usability and satisfaction of patients and doctors with the m-app.

### 2.6 Statistical analysis

Data on patient demographics and cancer treatment are presented as numbers and percentages. Symptom frequency and rates are presented as the means and standard deviations. Symptom duration and patient age are presented as medians and interquartile ranges. Comparisons between groups (patient reports versus doctor reports) were performed using the chi-square test. P<0.05 was considered statistically significant. For the EORTC QLQ C30 and the QLQ BR23, we followed the EORTC guidelines (23). In brief, for the functioning scales (range 0–100), higher values indicate better functioning, and for the symptom scales (range 0–100), higher values indicate worse symptoms. To compare the differences in functioning and symptoms at 3 months from baseline, we used paired Student’s t-test with 1000 bootstrap tests. Calculations and analyses were performed in Excel and SPSS v.22.

## 3 Results

### 3.1 Study patients

**Figure 2:**
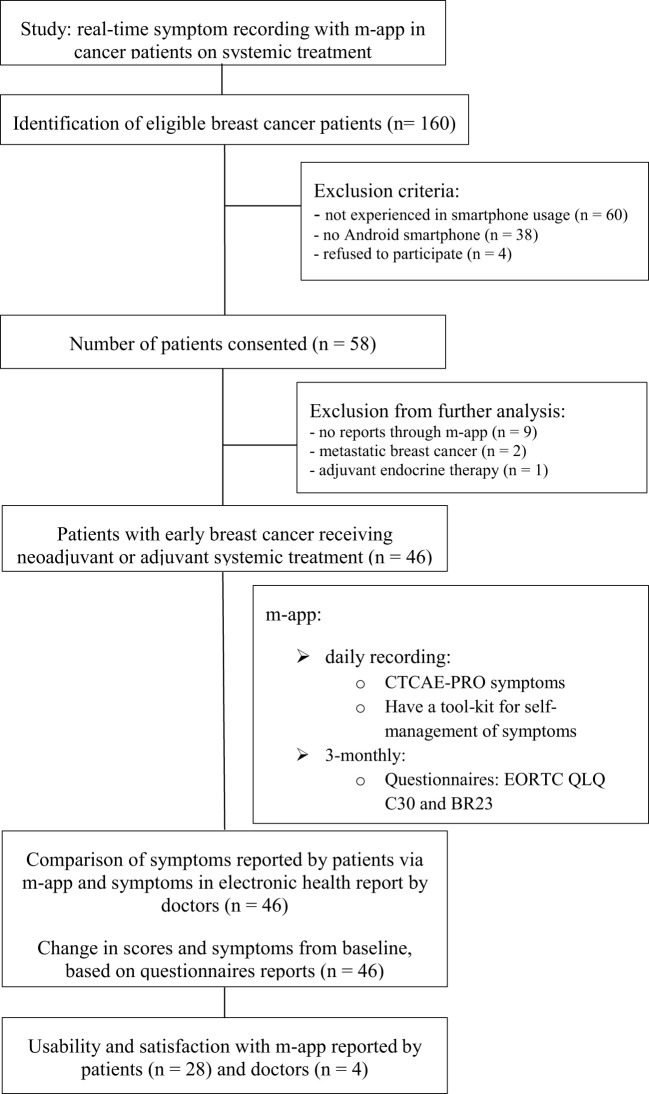
Study design and workflow diagram.

We approached 160 patients with early breast cancer. Sixty-four percent of patients were ineligible or did not consent. In the end, 58 patients signed an informed consent. It was later revealed that three patients did not meet the inclusion criteria (two were metastatic and one was not treated with chemotherapy). Nine of 55 (16%) eligible patients did not send any report and were also excluded from the study. Here, we present data on 46 female patients with early breast cancer who were treated with neoadjuvant or adjuvant systemic therapy. This represents 29% of the assessed population. **Figure 2** shows the consort diagram of the patients approached and included in the study and their study procedures.

The median age of the patients was 53.4 years (range 35–69 years). The demographic and tumor characteristics of the patients are presented in **Table 1**. Briefly, at inclusion, 58.7% of the patients had tumors > 20 mm in size, 32.6% had positive lymph nodes, and 63% had Grade 3 tumors. On the basis of immunohistochemistry, 60.9% of the tumors were luminal type A/B, 28.3% were HER2+ and 10.8% were triple-negative. Adjuvant treatment was delivered in 60.9% of patients, and 39.1% started neoadjuvant treatment. The most commonly used chemotherapy regimen was sequential treatment with anthracyclines and taxanes (65.2% of all patients). More than half of the patients (51.2%) underwent breast-conserving surgery, and 48.8% underwent mastectomy. In the mastectomy group, 19 of 22 patients underwent immediate reconstruction with a tissue expander or abdominal flap. Only 19.6% of patients had axillary dissection, and all others had sentinel node biopsy.

**Table 1:** Patients demographic and tumor characteristics.

Patients demographic and tumor characteristics		n (%)
Age	Median (25th, 75th quartile)	
	53.4 (46.2, 57.1)	
ECOG Performance status	0	34 (73.9)
	1	12 (26.1)
Histology n (%)	Nonspecial type	42 (91.3)
	Lobular	4 (8.7)
Grade	Grade 1	3 (6.5)
	Grade 2	14 (30.5)
	Grade 3	29 (63.0)
Ki-67	<20%	8 (17.4)
	≥20%	38 (82.6)
IHC based subtype	HR+/HER2-	28 (60.9)
	HER2+	13 (28.2)
	HR-/HER2-	5 (10.9)
Tumor stage	T1 (≤20 mm)	19 (41.3)
	T2 (21-50 mm)	19 (41.3)
	T3 (>50 mm)	6 (13.2)
	T4	2 (4.3)
Nodal stage	Node negative	31 (67.4)
	Node positive	15 (32.6)
Chemotherapy	Neoadjuvant	18 (39.1)
	Adjuvant	28 (60.9)
Type of chemotherapy	Anthracyclines &→ Taxanes	30 (65.2)
	Taxanes	11 (23.9)
	Anthracyclines	1 (2.2)
	TCHP	4 (8.7)
Breast tumor surgery	Mastectomy	22 (47.8)
	Breast conserving surgery	24 (52.2)
Breast reconstruction	Reconstruction with tissue expander	11 (23.9)
	Flap reconstruction	8 (17.4)
Axillary node's surgery	Sentinel lymph node biopsy	37 (80.4)
	Axillary dissection	9 (19.6)

HR, hormone receptor; HER2, receptor tyrosine-protein kinase erbB-2; TCHP, docetaxel + carboplatin + trastuzumab + pertuzumab; IHC, immunohistochemistry; ECOG, Eastern Cooperative Oncology Group.

### 3.2 Frequency and grades of symptoms

**Figure 3:**
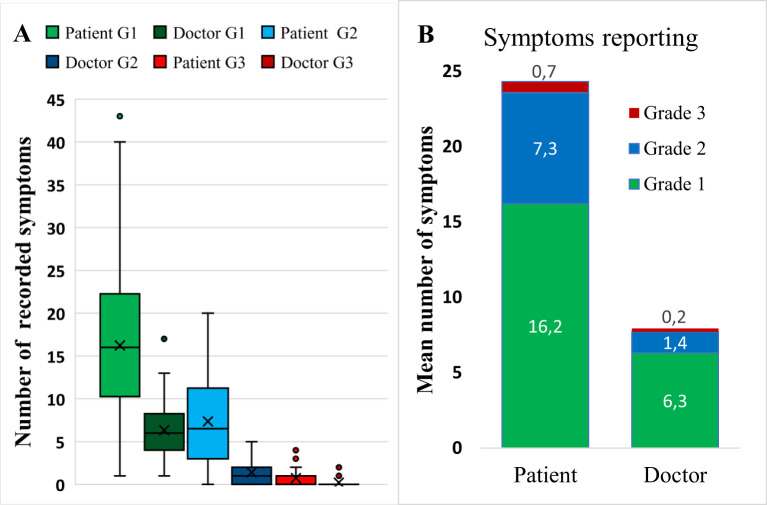
Number and grades of symptoms per patient according to maximal severity. **(A)** Comparison of Grade 1–3 symptoms reported by patients and doctors, presented as boxplots. Boxes represent quartiles 25 and 75 with the means (indicated by crosses) and medians (indicated by lines) inside the box. **(B)** Summarized mean number of symptoms by maximal grade assessed by patients and doctors. G1, Grade 1; G2, Grade; G3, Grade 2.

Over the entire study period, patients reported 75 different symptoms (minimum 4 and maximum 45). The median number of symptoms per patient was 25 (IQR 18–31.8). Among these, 16.2 (67%) had a maximum grade of 1, 7.3 (30%) had a maximum grade of 2, and 0.7 (3%) had a maximum grade of 3. Doctors observed 49 (minimum of 1 and maximum of 21) different symptoms; the median number of symptoms per patient was 7 (IQR 6–10). Among these symptoms, 6.3 (80%) were Grade 1, 1.4 (17%) were Grade 2, and 0.2 (3%) were Grade 3 (**Figure 3A**). There was a statistically significant difference in the reporting frequency of symptoms between patients’ and doctors’ reports (p ≤ 0.0001 for grades 1 and 2 and p=0.004 for grade 3), as shown in **Figure 3B**.

**Figure 4:**
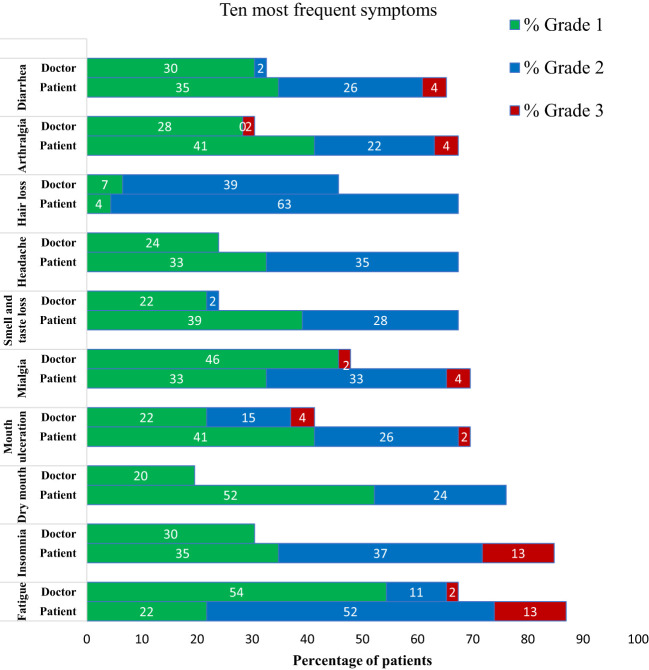
Comparison of the 10 symptoms most frequently reported by patients (daily via mobile application) and by doctors in electronic health records. Symptoms are presented as the percentage of patients experiencing these symptoms, with the corresponding severity expressed in grades 1–3.

On the basis of the m-app reports, the ten most frequently reported symptoms were fatigue, insomnia, dry mouth, ulcers of the oral mucosa, muscle pain, changes in smell and taste, headache, hair loss, arthralgia and diarrhea. These symptoms were experienced by 65–87% of all patients. The doctor’s reports of the frequencies and grades of symptoms were the closest to the patients’ reports with oral ulcers and hair loss. With respect to the other 8 symptoms, the doctor’s estimate was significantly lower in frequency and grade than the patient’s estimate was. The symptoms most underreported by doctors were insomnia and dry mouth (**Figure 4**). Six Grade 3 symptoms reported by patients are presented in **Figure 4**; in addition, abdominal pain, obstipation, dysuria, fever, skin rash and nail changes were reported as G3. The doctors reported 4 symptoms of G3, as presented in **Figure 4**, as well as polyneuropathy and vertigo.

### 3.3 Duration of symptoms

**Figure 5:**
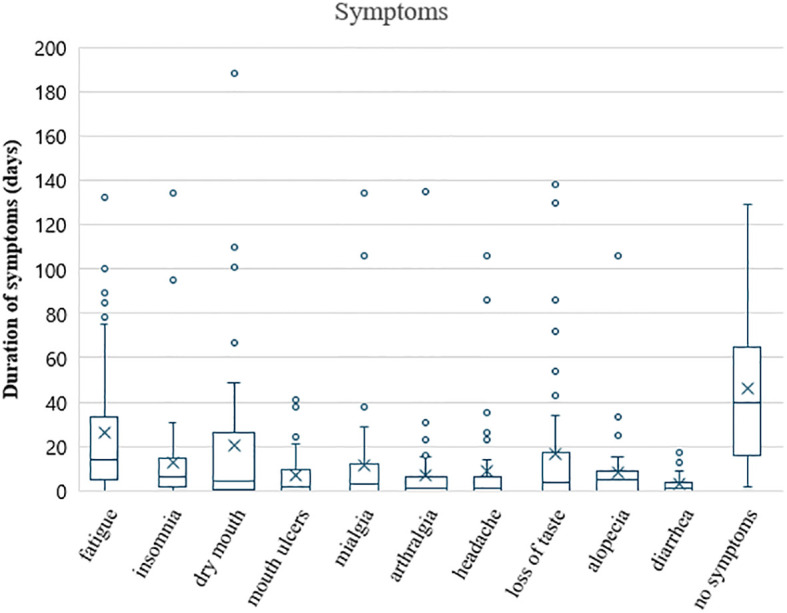
Duration of the 10 most frequent symptoms in patients on systemic therapy, presented in boxplots. Boxes represent the interquartile range with the mean (cross) and median (line) in the box, whiskers are to a maximum of 1.5 times the interquartile range, and points outside the whiskers represent outliers. The last boxplot represents the time without symptoms.

The mean time of recording symptoms in m-app was 115 days, ranging from 18–205 days. The cumulative duration of each of the 10 most often reported symptoms is presented in **Figure 5**. Fatigue, dry mouth and loss of taste had higher interquartile ranges than did the other factors. Except for fatigue and dry mouth, the median duration of each symptom was mostly short (less than 20 days). Furthermore, the period without symptoms was relatively long, accounting for 42% of the total duration of systemic therapy.

### 3.4 Changes in quality of life based on the EORTC C30 and BR23 questionnaires

At baseline, patients reported high scores (>75) on all five functional scales of the EORTC QLQ C30 and the EORTC QLQ BR23 for body image. However, their perception of global health status (GHS/QoL) was not high (53.1), and their future perspective was low (36.2). Symptom scores ≥ 20 were reported for insomnia (40.6), fatigue (34.3), pain (25.4) and breast symptoms (23.3). A detailed description of the functional and symptom ratings is provided in **Table 2**.

**Table 2:** Baseline patient-reported outcomes based on the European Organization for Research and Treatment of Cancer Quality of Life Core Questionnaire 30 (EORTC QLQ C30) and Breast Module 23 (EORTC QLQ BR23) and reports after 3 months of systemic treatment and changes from baseline (differences).

Module	Baseline Mean (SD)	3 months Mean (SD)	Difference Mean (SD)	95% CI	P value
EORTC QLQ C30 Global health status/QoL					
GHS/QoL	53.1 (22.7)	56.0 (22.2)	+3.2 (27.9)	-13.4; 7.0	0.524
Functional scales					
Physical functioning	89.0 (12.9)	81.2 (12.6)	-9.1 (14.9)	-14.3; -3.9	0.001
Role functioning	76.3 (28.5)	71.3 (19.3)	-9.2 (28.3)	-19.1; 0.7	0.067
Emotional functioning	78.9 (21.4)	77.2 (18.2)	-3.1 (19.5)	-9.9; 3.7	0.358
Cognitive functioning	87.0 (16.9)	78.7 (22.5)	-10.3 (17.8)	-16.5; -4.1	0.002
Social functioning	78.0 (25.4)	67.6 (23.1)	-10.7 (26.9)	-19.9; -1.4	0.025
Symptom scales/items					
Fatigue	34.3 (28.4)	53.3 (27.9)	+19.9 (32.5)	8.6; 31.3	0.001
Nausea and vomiting	10.1 (18.0)	7.8 (12.5)	-1.0 (16.9)	-6.9; 4.9	0.737
Pain	25.4 (31.0)	32.4 (31.2)	+10.3 (40.0)	-3.7; 24.2	0.143
Dyspnea	9.4 (16.7)	25.5 (26.3)	+13.7 (23.4)	5.6; 21.9	0.002
Insomnia	40.6 (36.5)	54.9 (42.5)	+12.7 (42.7)	.2.1; 27.6	0.091
Appetite loss	18.1 (27.9)	20.6 (27.2)	+2.9 (27.7)	-6.7; 12.6	0.54
Constipation	18.1 (29.6)	13.7 (29.7)	-3.9 (43.2)	-19.9; 11.2	0.6
Diarrhea	8.0 (17.5)	19.6 (34.0)	+13.7 (40.2)	-0.3; 27.8	0.055
Financial difficulties	15.9 (27.0)	21.6 (27.1)	+6.9 (25.7)	-2.1; 15.8	0.128
EORTC QLQ BR23 Functional scales					
Body image	84.1 (21.8)	73.3 (24.2)	-12.3 (21.8)	-19.9; -4.7	0.002
Sexual functioning	64.1 (31.0)	71.1(27.6)	+8.3 (21.0)	1.0; 15.7	0.027
Sexual enjoyment	62.3 (42.5)	72.5 (37.1)	+8.8 (34.3)	-3.1; 20.7	0.141
Future perspective	36.2 (35.0)	34.3 (38.0)	-1.0 (41.4)	-15.4; 13.4	0.891
Symptom scales/items					
Systemic therapy side effects	14.7 (13.9)	33.7 (18.6)	+19.6 (24.6)	11.1; 28.2	<0.001
Breast symptoms	23.3 (21.6)	11.8 (12.3)	-12.1 (22.7)	-20.0; -4.2	0.004
Arm symptoms	18.8 (23.0)	11.5 (14.2)	-7.4 (19.5)	-14.2; -0.5	0.035
Upset by hair loss	5.8 (20.1)	75.5 (36.1	+73.5 (19.5)	59.6; 87.5	<0.001

After 3 months of systemic treatment, patients reported significant deterioration in body image and physical, cognitive and social functioning; however, sexual functioning increased. Symptoms scales revealed significantly increased severity of some symptoms: hair loss for 73.5 points, fatigue for 19.9 points, systemic therapy side effects for 19.6 points and dyspnea for 13.7 points. On the other hand, their arm and breast symptoms significantly decreased. The GHS/QoL score remained stable (**Table 2**).

### 3.5 Satisfaction with the mobile application

**Figure 6:**
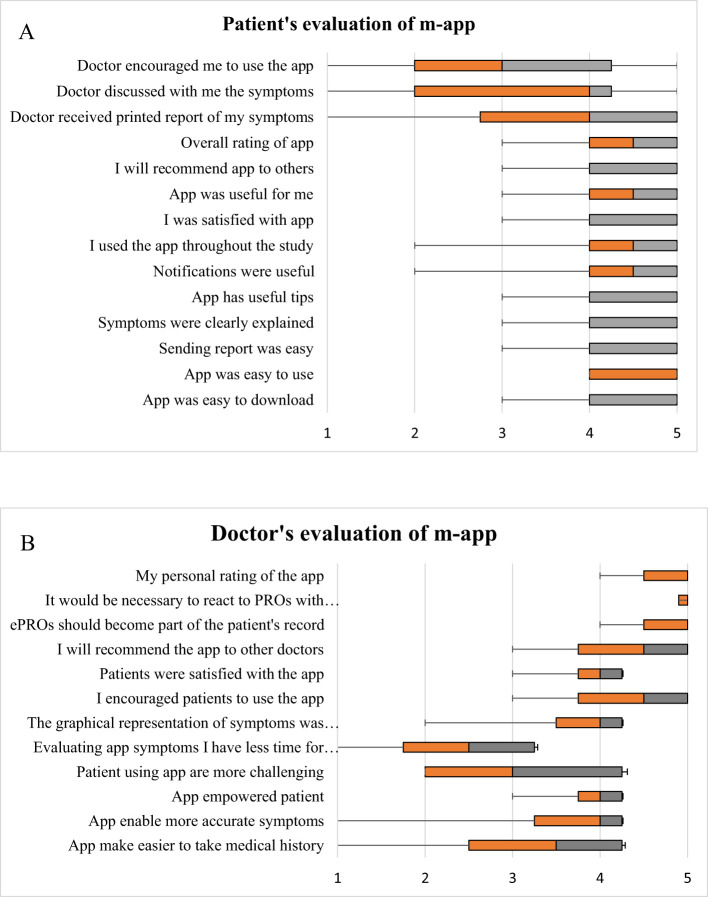
Evaluation of the mobile application (m-app) by patients **(A)** and by doctors **(B)**. The results are presented as boxplots and whiskers. The evaluation was performed on a 5-point scale, where 1 represents the lowest value or level of satisfaction and 5 represents the highest value or level of satisfaction. The orange part of the box represents quartile 25 to the median, and the gray part of the box represents quartile 75 to the median.

Six months after the end of the study, we collected anonymous feedback from both patients and doctors regarding the usefulness of and level of satisfaction with the m-app. Results are presented in **Figure 6**. A total of 28 patients (61%) and 4 doctors (44%) provided feedback. Among the 10 doctors who recruited patients, 4 doctors included only two patients, other the 6 doctors included 4–13 patients for participation in this study with ePROs. Patients and doctors rated the application with an overall score of 4.5 out of 5 (IQR 1.0). Patients generally remarked on applications’ ease of use and the included useful information and that they would recommend it to others. The IQRs for all these questions ranged from 4–5. The largest variance was in the answers to the questions of whether the notifications were useful and whether they used the application all the time. However, they were slightly less satisfied with the feedback from doctors. The doctors did not always have a printed report of the patients’ reported symptoms at the next check-up 4 (IQR 2.8–5). Patients rated feedback from doctors discussing e-reports and encouraging continued m-app usage even less favorably 3 (IQR 2.0–4.3).

Doctors were slightly less satisfied with the application, agreeing that it had useful tips for patients. However, they reported that it did not make it easier for them to take a medical history. Furthermore, they reported that it took them even more time and that patients were more demanding, so they would be less likely to recommend it to other doctors. Among the four that returned questionnaires, one expressed dissatisfaction, claiming the increased time required for patient examination and the impracticality of the application’s data for outpatient care. However, the remaining 3 doctors (75%) were satisfied with the application and did not consider it an additional burden during patient treatment. Notably, they mostly agreed that e-PRO information should be part of a patient’s EHR, but there should be warranted alerts on serious reports. 

## 4 Discussion

This prospective cohort study focused on daily symptom reporting in patients undergoing chemotherapy for early breast cancer. Patients using ePROs reported a significantly higher number of symptoms, and these symptoms were more severe than those reported in the subsequent assessment and interpretation by doctors during the same patients’ next outpatient visit. The symptoms most underreported by doctors were insomnia and dry mouth. We also used two validated questionnaires, the EORTC QLQ C30 and BR23, and compared periodic and daily symptom reporting. Patient and doctor satisfaction with and usability of the m-app were evaluated by patients and doctors. Both expressed high levels of satisfaction, although doctors expressed a need for a plan to manage severe symptoms when they were reported and a concern that they would be able to take over the additional workload to react to severe symptoms.

In the last decade, ePROs have become a hot topic due to advancements in digital technology and the widespread availability of smartphones, making these services globally accessible. The logical next step is the use of ePROs as a communication tool between patients and doctors, particularly in medical treatments such as cancer treatment, where prompt action at the onset of symptoms is crucial. The goal is to ensure the maintenance of patients’ QoL and adherence to their treatment regimens. Several small studies have been performed worldwide; however, the routine use of ePROMs and incorporation of these data into EHRs were still very rare until recently (11, 24, 25). The experience of The Christie NHS Foundation Trust, launched in 2019, stands out as a key example of large-scale ePROM integration into routine oncology care (26, 27). Real-time data entry into the EHR—without relying on separate platforms—enhanced the clinical usefulness for treating clinicians. Reported benefits include fewer hospital admissions, better quality of life, potential survival benefits, and more efficient consultations (26, 28). Later evaluations identified barriers among older patients with limited access to technology and those receiving radical treatment. Clinicians have also requested clearer data displays within EHR (27). Another UK oncology center that uses ePROMs for patients receiving targeted and immunotherapies has shown a reduced need for face-to-face visits (29). A program using the PRO-CTCAE and pain scales during palliative radiotherapy offered automated self-care guidance and guidance for contacting healthcare providers (30). Studies with ePROM implementation highlight benefits such as improved symptom control, patient-provider communication and quality of life (30–32). The European Society for Medical Oncology (ESMO) now recommends PROM-based monitoring after cancer treatment, including near end-of-life care (33).

In this trial, we prospectively assessed daily symptom reports. Over 4–6 months of chemotherapy, patients reported 75 symptoms (median 25 per patient): 67% mild, 30% moderate, and 3% severe, with fatigue, insomnia, stomatitis, myalgias, arthralgias, and diarrhea among the severe symptoms. Our findings are consistent with an Italian study that also identified fatigue as a highly prevalent but often overlooked severe side effect of adjuvant systemic therapy (12). We then compared patient-reported symptoms with doctor assessments at regular check-ups. Doctors reported fewer symptoms (49 *vs*. 75, median 7 per patient, IQR 6–10) and generally graded them lower (**Figure 3**). However, Grade 3 symptoms accounted for 3% of the symptoms in both reports. Patients most frequently reported fatigue, insomnia, and dry mouth (**Figure 4**), with insomnia and dry mouth being symptoms the most underreported by doctors. Our findings align with those of previous studies (9, 34) showing that doctors tend to underestimate symptoms, particularly those where specific treatments are unavailable. Notably, Grade 3 muscle/joint pain, fatigue, and nausea were underestimated (35). Overall, our results suggest that patients report numerous adverse events affecting their QoL. In contrast, doctors prioritize reporting those that influence treatment decisions, such as not prescribing (postponing) therapy, dose adjustments, or supportive therapy adjustments. We acknowledge the potential for assessment bias in the doctor’s grading of symptom, given the retrospective nature of the assessment. The risk was to some extent alleviated by the use of single assessor. While it may enhance consistency, it does not eliminate bias and could introduce a single-assessor bias, where that an individual’s interpretation systematically influences the data.

In addition, our study highlights the effectiveness of the m-app in managing adverse symptoms. The low percentage of Grade 3 symptoms is likely due to the supportive measures provided by the m-app toolkit, which helps patients self-manage mild to moderate symptoms, preventing escalation to Grade 3. This aligns with previous studies showing that PROs enable early recognition and management of adverse events, reducing symptom duration (12, 36). Notably, our patients were symptom-free for 42% of the treatment duration, whereas Antonuzzo et al. (12) reported a symptom-free period of only 15%, likely due to differences in patient characteristics, chemotherapy regimens, or study methodologies. In our previous prospective cohort study, we demonstrated that early breast cancer patients using the m-app toolkit experienced improved quality of life (QoL) both in the first week after chemotherapy and at the end of treatment compared with the control group without application support (21). Similarly, Fjell et al. reported that an interactive application reduced symptom burden and improved emotional well-being during neoadjuvant chemotherapy for patients with breast cancer (37).

There is a worldwide debate regarding the optimal frequency of ePRO assessment during cancer therapy: once-weekly or daily assessment. Both approaches have advantages and limitations, with more precise symptom reporting in daily monitoring and less burdensome reporting weekly. Symptom monitoring empowers patients to actively contribute to problem-solving measures. On the other hand, experiences revealed survey fatigue in patients, which means that they responded less regularly when they became familiar with symptom management. Therefore, strategies on the frequency of symptom recording should be adjusted to a particular setting (active chemotherapy treatment, maintenance treatment, follow-up on chronic therapy) (38). For example, during adjuvant endocrine therapy, 73.2% of patients completed the baseline survey, whereas 69.6% participated in at least one 3-monthly ePRO follow-up survey via a smartphone within the first six months (39). Like our study, Daly et al. utilized daily symptom reporting in adults receiving anticancer therapy. They analyzed the impact of remote monitoring of red alerts (severe symptoms) and reported that 8.7% of patients who triggered a red alert required an acute care visit within seven days, whereas 2.9% of those without a red alert required an acute care visit. However, after six months, only a quarter of the patients continued daily reporting. The most common triggers for red alerts are pain, dyspnea, and functional decline (40).

A key consideration is whether real-time symptom reporting, despite its complexity for both patients and healthcare professionals, has advantages over cross-sectional validated questionnaires such as the QLQ-C30 and BR23. In our study, real-time reporting identified mouth ulcers as the 4th most prevalent symptom (**Figure 4**), and questions regarding this particular symptom are not available in either C30 or BR23. However, the updated version of BR23, BR42, now includes questions addressing mouth ulcers under items 57 and 58 (soreness and redness in the mouth). Among the 10 most common symptoms reported by our patients, three (dry mouth, taste changes, and hair loss) are covered in BR23, whereas the remaining six are included in C30. Notably, BR23 does not score symptoms individually but rather as part of a composite “systemic therapy side effects” score. Additionally, the timeframe of assessment differs: C30 evaluates symptoms over the past week, whereas BR23 covers the past four weeks. In our view, daily symptom reporting has advantages at the start of a new treatment or for treatments where several side effects are expected, allowing for immediate intervention, whereas C30 and BR23 provide a more comprehensive overview of baseline status, long-term symptom trends over milestones and monitoring adherence to treatment.

Despite the proven benefits, the widespread integration of e-PROs into routine clinical practice faces numerous challenges, including time consumption, logistical issues regarding severe symptom management and physician reluctance to adopt new technologies. With respect to adoption by doctors, four of our doctors included only two patients in the study, other the six included 4–13 patients. In our study, 56% of the doctors chose not to provide feedback on their evaluation and satisfaction with the application. Among the 44% who did share their opinions (4 doctors in total), the majority (75%) expressed satisfaction and supported its integration into daily practice. However, one doctor (25%) provided a strongly negative opinion, viewing the application as a burden that detracts from the valuable time spent with patients without offering significant additional benefits. There was some additional time spent on patients’ check-ups. The initial patient enrolment took an additional half hour from the physician, and the first follow-up visit was extended by approximately 5–10 minutes for report review and app discussion. Subsequent consultation times were not prolonged, as symptom history taking was potentially made more efficient.

In contrast, patients expressed a very positive opinion on the application. They saw it as a tool that provides a sense of security and helps them manage side effects, which is in line with the observations previously reported in different studies and meta-analyses (11, 41–43). This positive perception is reflected in high initial adherence to adverse effect reporting, which gradually declines as patients become more proficient in managing side effects. Similarly, Handa et al. reported a 25% decrease in adherence to m-app usage from the first chemotherapy cycle to the fourth cycle (35). Lee et al. reported that patients with greater expectations of the application’s usefulness and ease of use were more likely to adopt it, although ease of use did not directly impact compliance (39). We are aware, that usability and satisfaction survey feedback was received from 28 of 46 patients (61%), which represents attrition bias. Owing to survey anonymity and postal distribution, we could not increase the percentage of respondents using reminders, causing attrition bias. The delay in performing the survey (6 months poststudy) may have contributed to nonresponse, possibly introducing selection bias—more satisfied users may have been more likely to respond. Nevertheless, frequent users likely provided the most informed feedback. When considering the usability and satisfaction of m-app users, we need to be aware of the critical limitations of our study in terms of lack of representation from older, digital or health-literacy marginalized populations. On the basis of our sample of young and digitally literate women with breast cancer, it is difficult to draw conclusions about the generalizability of the findings to the general population. Among the physicians, 4 out of 10 responded. Nonrespondents may reflect resistance to use ePROMs, as they had not adopted them in routine practice. This may partly explain also the observed difference in symptom reporting grades.

Considering all the advantages and barriers, we believe that ePROs are promising to become an integral part of routine patient assessment in the near future. Only a quarter of the surveyed practitioners reported capturing PROs in routine clinical practice (44). Many countries have experience with various cancer applications, but only a few enable real-time recording and sharing of ePROs with clinicians (26, 29, 30, 45, 46). Despite the known advantages, the acceptance of ePROs in routine clinical care has been hindered by work processes and technology-based challenges (18). Clinic staff reported frustration and dissatisfaction, citing increased workload without perceived benefits for oncological treatment (11). Further investigation is needed to integrate these applications with EHR, emphasizing the importance of implementation standards, certification and user feedback. The inclusion of stakeholders, health professionals, IT specialists, and patient representatives is crucial in the process (47–50). This multidisciplinary approach could improve patients’ perceived health, outcomes, and hospital resource optimization for ePROs. The benefits of remote control of symptoms include proactive early intervention to reduce symptoms, symptom self-management and improved patient–provider relationships (38).

Our study has several strengths and limitations. One of the major strengths, in our opinion, is daily symptom reporting via the m-app, as only a few studies have enabled real-time symptom monitoring thus far. Additionally, our study empowers patients with appropriate tips for self-management of mild to moderate symptoms. Furthermore, it also offers valuable insights into the different perceptions of adverse effects between patients and doctors, as well as their opinions regarding PRO usage. The main limitations, on the other hand, are the small number of participants, the single-center nature of the study and the requirement for smartphone access, which excluded a significant portion of the elderly population. We acknowledge the temporal mismatch between patients’ daily reporting and retrospective grading of doctors’ symptom assessment as a potential confounder. Next, we did not anticipate creating alerts for Grade 3 symptoms; instead, following our usual practice, we instructed patients to visit the nearest emergency department. There were also some increased costs regarding staff engagement (patient education and preparing reports for doctors). Finally, we used our own questionnaire to evaluate the level of satisfaction and usability of the m-app; however, we later discovered that a validated usability questionnaire, the mHealth App Usability Questionnaire (MAUQ), already exists (51). Therefore, we have validated this questionnaire for the Slovenian language for future studies with the m-app.

## 5 Conclusion

Our study demonstrates that real-time symptom tracking via an m-app improves symptom detection, with patients reporting nearly twice as many symptoms as doctors do. While patients found the application highly useful (4.5/5 rating), doctors were more hesitant, perceiving ePROs as an additional burden. Despite some reluctance, mobile-based ePRO follow-up is promising, but requires more systematic, inclusive, and scalable implementation studies to establish feasibility and sustainability in routine practice.

## Data Availability

The raw data supporting the conclusions of this article will be made available by the authors, without undue reservation.

## Supplementary material

Supplementary Figure 1 Free text presentation of patient reported symptoms.

Supplementary Figure 2 Graphical presentations of time-course of patient reported symptoms and their grades.

Supplementary Figure 3 Patient reported symptoms in Excel table with their grades.
