# Supplementary figures and images for: Patients’ daily reporting of symptoms via mobile application reveals a significant difference between patients’ perceptions and doctors’ interpretations
Source: Front Oncol. 2025 Jul 8;15:1595322. doi: 10.3389/fonc.2025.1595322 (PMC12305432; doi:10.3389/fonc.2025.1595322)

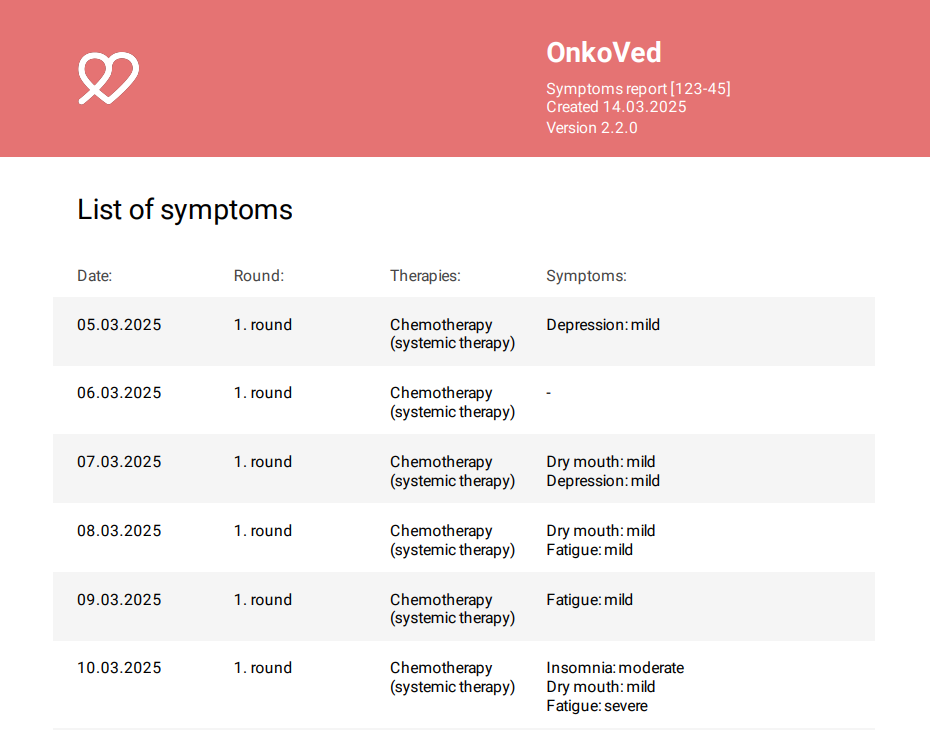

Supplement: Supplementary Figure 1 — Free text presentation of patient reported symptoms. [file Image1.png]

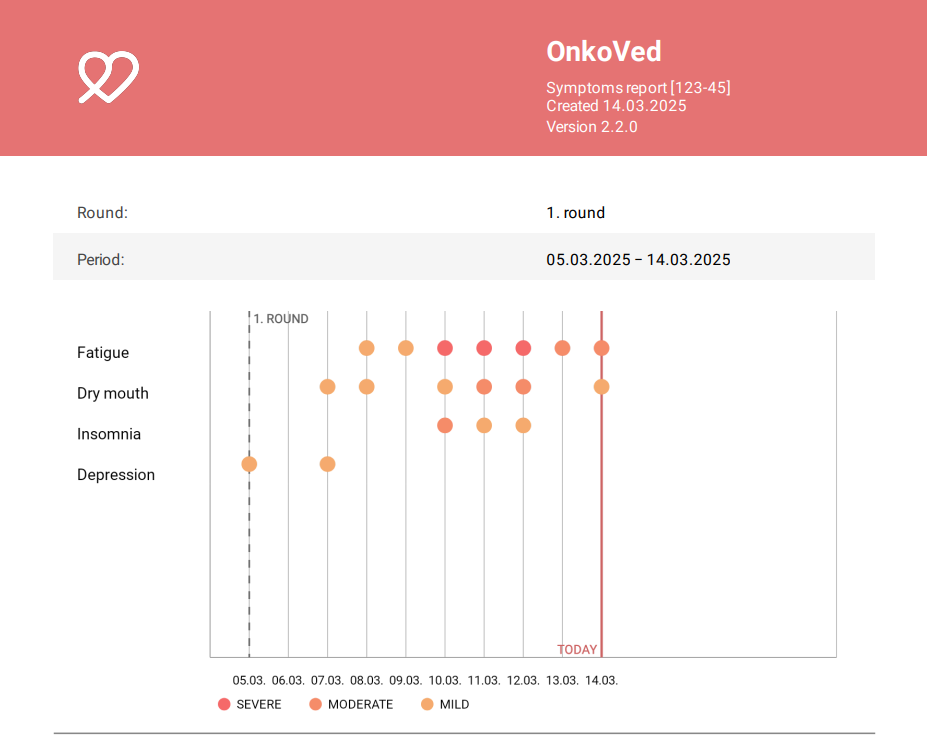

Supplement: Supplementary Figure 2 — Graphical presentations of time-course of patient reported symptoms and their grades. [file Image2.png]

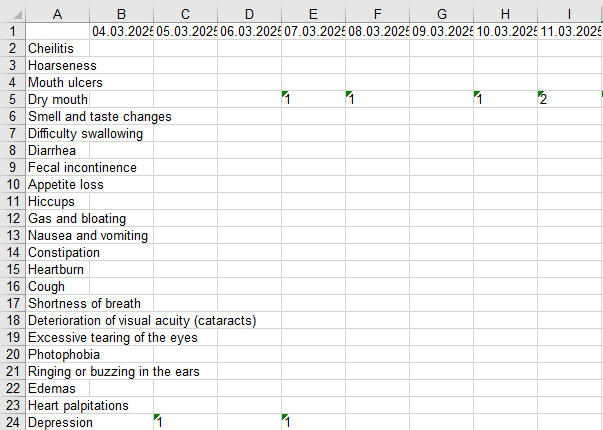

Supplement: Supplementary Figure 3 — Patient reported symptoms in Excel table with their grades. [file Image3.png]
